# The existence and potential of germline stem cells in the adult mammalian ovary

**DOI:** 10.1080/13697137.2018.1543264

**Published:** 2019-01-02

**Authors:** E. E. Telfer, R. A. Anderson

**Affiliations:** a Institute of Cell Biology, School of Biological Sciences, University of Edinburgh, Edinburgh, UK;; b MRC Centre for Reproductive Health, Queen’s Medical Research Institute, University of Edinburgh, Edinburgh, UK

**Keywords:** Oogonial stem cells, ovarian regeneration, oocyte, neo-oogenesis

## Abstract

It has long been accepted that the complement of follicles within the ovary is formed before birth in humans, or shortly after birth in rodents, and that no follicles are formed thereafter. This follows entry of all oogonia into meiosis in fetal life, with no remaining germ stem cells in the ovary, in contrast to the presence of spermatogonia in the testis. This has been brought back into debate in recent years, following the demonstration of isolation of cells expressing both germline and stem markers from the postnatal ovary in several species, including humans. We describe these cells as putative ovarian stem cells. Isolation of these cells is challenging, adding to the debate as to their existence, and the validity of DDX4 as the main marker used for their isolation has also to be questioned. While different groups have used varying techniques and indeed terminology to describe these cells, the body of evidence regarding their initial characterization after isolation is growing. There remain very limited data regarding their developmental potential, but the demonstration of the production of functional oocytes from induced pluripotent stem cells and the advances in ovarian follicle culture techniques provide a basis for such studies.

## Chinese abstract

长久以来我们都认为人体内的卵泡在出生前形成, 啮齿类的卵泡在出生后不久形成, 之后并不会增加。胎儿期所有卵原细胞进入有丝分裂后, 卵巢内没有剩余的生殖干细胞, 对比来讲, 而睾丸内则存在精原细胞。近年来, 随着从包括人类在内的一些物种在出生后的卵巢里分离出同时表达生殖系和干细胞标记的细胞, 这又引起了争论。我们把这些细胞假定为卵巢干细胞。分离这些细胞比讨论他们是否存在更有挑战性, DDX4----作为判断细胞是否被分离出的主要标志物, 也同样被质疑。虽然不同的研究小组使用了不同的技术和术语来描述这些细胞, 但有关分离后这些细胞最初特征的证据越来越多。关于多能干细胞发育潜能的资料非常有限, 但是从诱导多能干细胞中产生功能性卵母细胞的证据和卵巢卵泡培养技术的进展为此类研究提供了基础。

## Introduction

**Figure 1.**
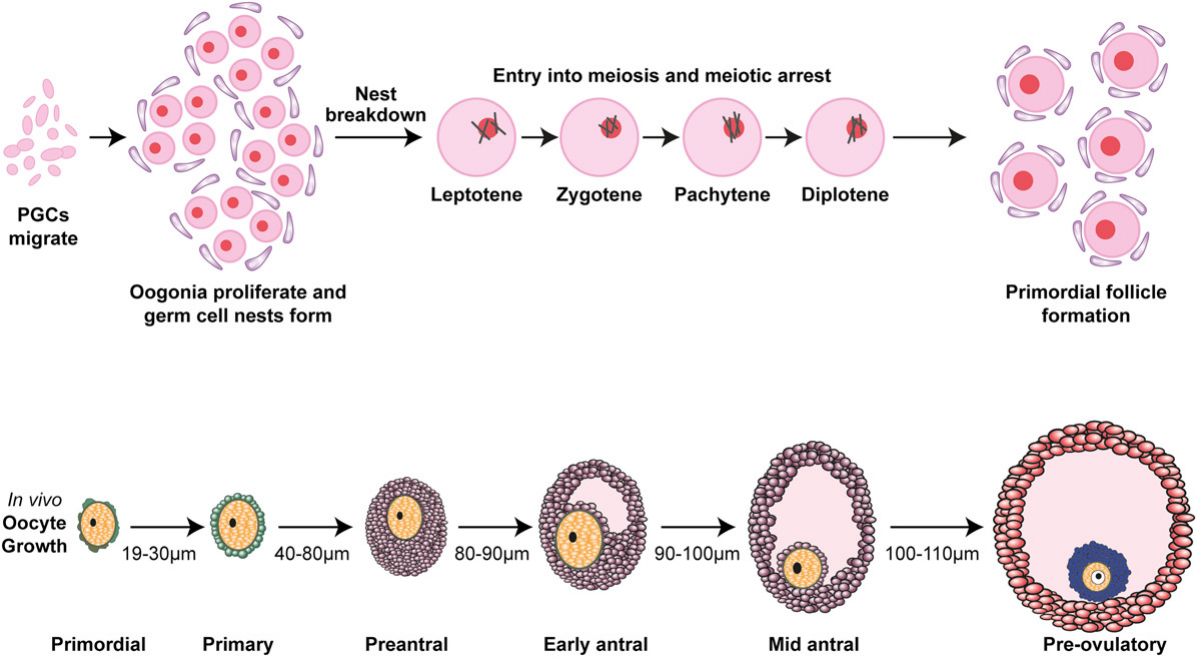
Schematic of the key stages of oocyte maturation. Upper panel: proliferative primordial germ cells (PGCs) form nests and then enter meiosis during fetal life. Nest breakdown results in the formation of primordial follicles. Lower panel: after growth activation, follicles progress through the stated phases, with the oocyte ultimately reentering meiosis and being released from the follicle. Sizes given are of the oocyte, not the follicle, showing how it also grows dramatically during this process. Reproduced from Anderson and Telfer^1^ with permission.

The mammalian ovary is a highly dynamic organ that undergoes many structural and functional changes as it fulfills its two major roles of producing female gametes and the synthesis of sex steroids. In the human ovary, germ cells (oocytes) are formed during fetal life and they are enclosed within somatic cells (granulosa cells) to form primordial follicles. The primordial follicles consist of an oocyte, arrested at the diplotene (dictyate) stage of prophase I of meiosis, surrounded by a few flattened somatic cells (granulosa cells). For many years it has been assumed that there is a limited period during which oocytes can be formed and that the adult ovary has no capacity for germ cell renewal, and therefore primordial follicles represent a pool of oocytes that must last the woman throughout her reproductive lifespan (Figure 1).

The management of ovarian insufficiency and failure, including infertility caused by aging or damage, is largely based on the belief that the entire germ cell (oocyte) pool is present by the time of birth and that ovaries lose their capacity for oocyte renewal (oogenesis) under physiological and perturbed conditions. Whether the adult mammalian ovary has any capacity to undergo postnatal germ cell renewal has been the subject of debate for almost a century^2^. The consensus since the 1950s has been that the population of primordial follicles is fixed before or around the time of birth, depending on the species^3^. Although exceptions have been recognized; the existence of a continuous germline lineage has been identified in the ovaries of prosimian primates for many years^4^. The existence of similar cells in the ovaries of adult human ovaries had not been actively studied until recently.

The publication of a study proposing germ cell renewal in adult mice^5^ reopened the debate on germ cell renewal and has led to several studies in a number of mammalian species that have identified cells with potential for germ cell development. The isolation and identification of oocyte-producing germline cells, in ovaries of adult mammals in general, and humans in particular, remained elusive until 2009 when putative germline stem cells were isolated from adult mouse ovaries^6^ and then subsequently from human ovaries^7^. However, opinion remains divided with regard to the existence, significance, and derivation of these putative germline stem cells. Critics have argued that experimental techniques, data analysis, and interpretation are flawed in studies that have identified putative female germline stem cells, and that *in vitro* expansion could explain the presence of germline markers in mitotically active cells derived from adult mammalian ovaries. Many of the early criticisms have been addressed by further studies but it is clear that the isolation of these cells is difficult, with several groups unable to identify them. This review will focus on the evidence available to support the existence and potential of putative germline stem cells in the adult mammalian ovary, focusing particularly on humans.

## Isolation and identification of cells

Whilst this remains a controversial area, there is now a large body of experimental evidence that demonstrates the existence of cell populations with molecular characteristics consistent with germline cells within the adult ovary of several mammalian species, including humans. The physiological significance of these cell populations still needs to be clarified but, given that adult stem cells have now been identified in most organ systems, it would seem likely that the ovary contains stem cells for all component cell types^8^.

Central to the identification of these cells is their ability to undergo mitotic division, to express proteins associated with the germline, such as DDX4, KIT, DPPA3, IFITM3, and PRDM1, and with pluripotency, including POU5F1, LIN28, and NANOG. Several studies have now demonstrated the isolation of mitotic cells expressing germline markers from ovaries of adult rodents^6^ ^,^ ^7^ ^,^ ^9–11^, cows^12^, sheep^13^, primates^14^, and humans^7^ ^,^ ^12^ ^,^ ^13^ ^,^ ^15–18^. These cells have been called female germline stem cells, oogonial stem cells (OSCs), egg precursor cells, or indeed very small embryonic-like stem cells. In this review the cells will be referred to as putative OSCs in recognition that these cells have yet to be fully characterized and their true stem cell potential determined.

**Figure 2.**
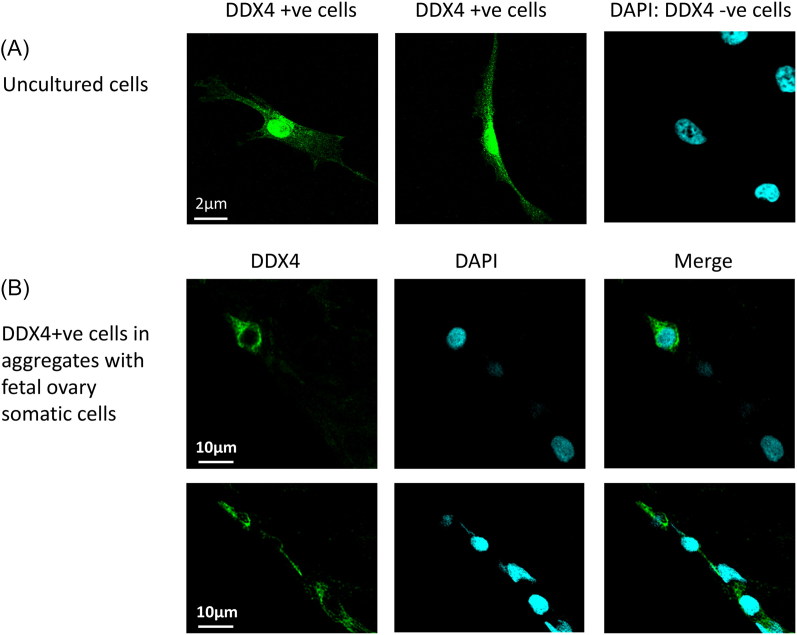
(A) Immunocytochemistry of DEAD box polypeptide 4 (DDX4) expression in freshly sorted human putative oogonial stem cells (‘uncultured cells’), with 4′,6-diamidino-2-phenylindole (DAPI) staining of DNA in DDX4-negative cells (centre panel, and merged image (right panel). (B) DDX4 and DAPI staining of putative oogonial stem cells after culture in aggregates of human fetal ovarian somatic cells. Methods as described in Clarkson et al.^15^.

The populations of cells that have been isolated by different groups may not represent a homogeneous group. The emerging evidence suggests that there are different populations of putative stem cells within the adult ovary, some with germline characteristics and others with somatic cell characteristics. Isolation of putative OSCs has been based on the expression of the RNA helicase DEAD box polypeptide 4 (DDX4), which within the ovary is found only in the germline, using fluorescence-activated cell sorting (FACS) or magnetic-activated cell sorting. Isolated cells are then analyzed to determine the presence of other germline and stem cell markers. The reliance upon DDX4 as a marker to isolate these cells has attracted criticism^19–21^. Much of this criticism has centered around the assumption that DDX4 is only an intracellular protein and does not have a surface epitope^22^. DDX4 is expressed in the cytoplasm of oocytes and not on the surface but it has been found to be localized to the nucleus, cytoplasm, or membrane bound in a range of tissues and cells, with potentially dynamic localization depending upon the cellular context^23^. This is contrary to the assertion that DDX4 cannot be expressed on the cell surface. Recently published data from our laboratories showed that populations of cells expressing DDX4 can be isolated from adult human ovary using commercially available antibodies to DDX4, and clearly demonstrate that DDX4 can be detected on the surface of the freshly isolated cells (Figure 2)^15^.

**Figure 3.**
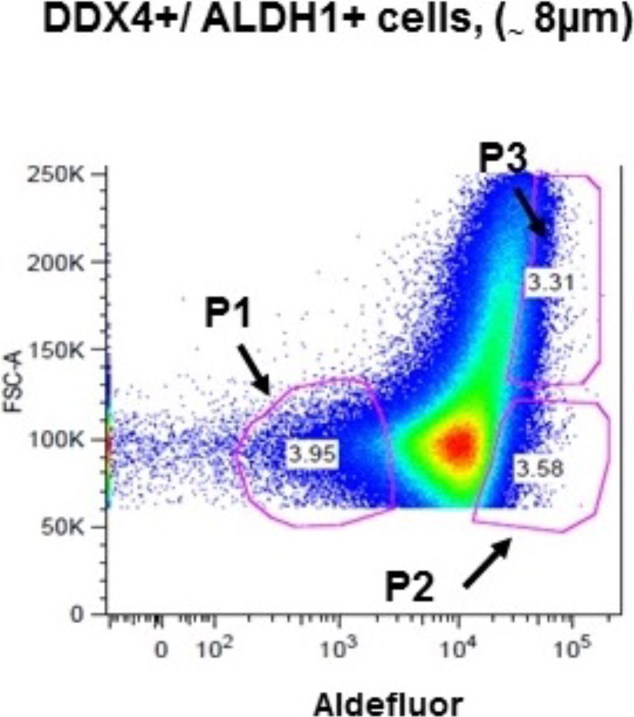
Flow cytometry plot illustrating selection of subpopulations of sorted ovarian cells based on cell size, presence of DEAD box polypeptide 4 (DDX4), and intensity of aldehyde dehydrogenase 1 (ALDH1). Methods as described in Clarkson et al.^15^. Populations 1 and 2 (P1, P2) are of similar size but differ in the degree of ALDH1 intensity and expression pattern. Populations 2 and 3 (P2, P3) have similar intensities of ALDH1 and DDX4 but cells in population 3 are significantly larger. FSC, forward scatter.

That several groups have been unable to isolate these cells or detect DDX4 expression is probably indicative of technical issues associated with cell dissociation and sorting, particularly from the human ovary which is much tougher than the rodent ovary with which most investigators in the field are more familiar. It is clear that the cells are sensitive and harsh dissociation methods may result in excessive cell death. The technique reported by Clarkson et al.^15^ describes a gentler modified dissociation method than that originally described by the Tilly group,^7^ which results in high cell survival prior to FACs. This refined methodology used a widely recognized marker of viable stem cells, aldehyde dehydrogenase 1 (ALDH1)^24^, in combination with antibodies against the external C-terminus of DDX4 for FACS of dissociated human ovarian cells. By combining these two markers, cells positive for DDX4 with varying degrees of stem cell capacity could be identified. The addition of this ALDH1 marker facilitates the isolation and identification of potential subpopulations of putative OSCs (Figure 3)^15^.

## Development of putative OSCs into oocytes

Putative OSCs are characterized by expression of primitive germ cell-specific and stem cell markers^7^ ^,^ ^15^ ^,^ ^25^. Isolated putative OSCs express germline markers (DDX4, KIT, DPPA3, IFITM3, and PRDM1) and pluripotency markers (POU5F1, LIN28, and NANOG), but not oocyte/follicle markers (CYP19A1, HDAC6, or ZP3). Transplantation of *in vitro* expanded green fluorescent protein-labeled mouse putative OSCs back into ovaries leads to the generation of fertilization-competent eggs that produce viable embryos and offspring^6^. Cells with similar characteristics have been isolated from adult human ovaries^7^ ^,^ ^15^ ^,^ ^18^. Cultured putative OSCs maintain a germline profile while actively dividing and have been shown to form oocyte-like structures *in vitro* after prolonged culture^7^ ^,^ ^18^ ^,^ ^26^ or short-term culture^15^ ^,^ ^18^. Fluorescently labeled human putative OSCs have generated green fluorescent protein-positive oocyte-like cells enclosed in host somatic cells, as assessed by morphology and expression of oocyte-specific markers after injection into adult human ovarian cortical tissue and xenotransplantation into an immune-deficient mouse for 7 days^26^. The formation of oocyte/follicle-like structures under these conditions has been explained as an *in vitro* transformation and not indicative of the true potential of the isolated cells. However, recent studies have demonstrated that freshly sorted human cells, analyzed without *in vitro* culture, express both germline and stem cell markers^15^ ^,^ ^18^. Furthermore, oocyte/follicle-like structures can be formed from freshly isolated human putative OSCs when combined with human fetal ovarian somatic cells to form aggregates during a culture period of 7 days^15^. Therefore, the developmental potential of the isolated cells cannot be dismissed as being an *in vitro* artifact.

Recent work has identified subpopulations of human putative OSCs based on their gene expression profile^15^. This may be significant in terms of developmental potential as they show some parallels with normal human germ cell developmental stages^27^. Sorting of cells using DDX4 and ALDH1 identified two distinct populations of cells of similar size (P1, P2) characterized by the presence or absence of the RNA binding protein Deleted in azoospermia-like (DAZL). DAZL is a major regulator of germ cell differentiation and essential for progression into meiosis^28^ ^,^ ^29^. DAZL is present in the P2 population but not in P1. Human studies have shown that DAZL expression is significantly upregulated in germ cells around the time of meiotic entry^27^, thus the cells collected in P2 may represent a more differentiated population that have greater potential to form functional gametes if given the right support^15^. The identification of subpopulations of putative OSCs could account for the lack of uniformity between results presented by investigators seeking to isolate a standardized single population of cells by FACS^22^ ^,^ ^30^.

Identifying and isolating cells with germline potential from the human ovary represents a major advance and could have many practical applications for human fertility, most clearly in assisted reproduction and fertility preservation/restoration. In order to make progress, it is essential to characterize fully the isolated cell populations that various groups have identified and to reach a consensus as to whether the cells are indeed stem cells or unipotent progenitor cells. The ‘oocyte-like’ cells derived from putative OSCs *in vitro* require somatic cell support and development of paracrine and junctional communication to form follicles if functional oocytes are to be produced. Developing these ‘oocyte-like’ cells *in vitro* will require developmentally appropriate culture systems including systems that support oocyte development from the earliest stages. If functional oocytes could be derived from putative OSCs and developed *in vitro*, this would indeed widen options for fertility preservation and restoration.

## Supporting oocyte formation from putative OSCS and growth *in vitro*

Complete oogenesis has been achieved *in vitro* starting from induced pluripotent stem cells derived from mice^31–34^. These studies clearly demonstrate the crucial role of somatic cells in supporting germ cell development from these earliest stages. Recapitulating this process *in vitro* using human-derived induced pluripotent stem cells has been much more problematic and so far can only reach the stage of oogonia (Science paper Saitou group).^32^ In contrast, isolated human putative OSCs form oocyte/follicle structures *in vitro* and after transplantation *in vivo* ^7^ ^,^ ^15^. The ability to obtain formation of follicles/oocytes from these cells enables characterization studies to be carried out. Expression patterns of factors required for early oocyte development as well as factors associated with entry into meiosis need to be determined, as well as detailed sequencing of populations of cells isolated from the adult ovary. Alongside characterization studies, the challenge will be to determine whether functional oocytes can be obtained from putative OSCs and this will require the development of defined culture systems. Systems that support the growth and development of immature human oocytes *in vitro* have been developed^35^ ^,^ ^36^ and will be invaluable for this work, although requiring optimization.

The concept of growing immature oocytes *in vitro* has been the subject of a great deal of research for almost 30 years. Complete growth *in vitro* from the most immature oocytes (primordial stages) with subsequent *in vitro* fertilization and production of live young has been achieved in mice^37^ ^,^ ^38^. Early work on this two-step culture system resulted in only one live offspring being obtained and this mouse had many abnormalities as an adult^37^. Following improvements in the technique and after modifications to the culture medium, several mouse embryos and offspring were obtained from *in vitro* grown oocytes combined with *in vitro* maturation and *in vitro* fertilization ^38^. This work has provided a proof of concept that complete oocyte development can be achieved *in vitro* and has driven the development of culture systems that could be applied to other species, particularly humans. The multistep culture systems that support the growth and development of immature human oocytes *in vitro* from primordial follicles right through to fully grown oocytes capable of meiotic maturation to metaphase II^36^ provide a robust and extensive *ex vivo* test of the potential of any oocyte-like cells that are formed from putative OSCs.

## Summary

There now exists a body of experimental evidence that supports the existence of cells with molecular characteristics consistent with germline progenitor/stem cells within the ovaries of a range of species, including in adult women. Different methods of isolation and characterization have been used and different terminologies exist to describe these cells, leading to some confusion. Ongoing research is continuing in several laboratories to further define and characterize cell types within the adult human ovary. The potential physiological relevance of these cells to adult ovarian function and fertility remains unknown, but their existence raises questions about why they do not appear to contribute to postnatal follicle formation, whether that is in fact correct, and if, how, and when their potential could be harnessed. Whilst there remains controversy over the biological significance of these cells, it must be acknowledged that their identification and isolation represent a significant advance with the potential to change infertility treatments, and possibly even the non-reproductive consequences of the loss of ovarian function, in the future.
